# A descriptive analysis of sporadic Creutzfeldt-Jakob cases in Vietnam: 31 patients from four tertiary care centers

**DOI:** 10.1016/j.prdoa.2025.100351

**Published:** 2025-05-30

**Authors:** Khoi Hong Vo, Nga Thi Bui, Dung Thi Hoang, Thanh Vinh Nguyen, Binh Thanh Nguyen, Tai Ngọc Tran, Anh Hai Nguyen, My Thi Le, Ha Thi Do, Thuan Duc Nguyen, Hai Van Ta, Toan Van Phan, Phuc Duy Phan, Thang Xuan Pham, Frank Xing, Daniel Truong

**Affiliations:** a Neurology Center, Bach Mai Hospital, Hanoi, Vietnam; b Department of Neurology, Hanoi Medical University, Hanoi, Vietnam; c Department of Neurology, VNU-University of Medicine and Pharmacy, Hanoi, Vietnam; d The Truong Neuroscience Institute, Orange Coast Memorial Medical Center, Fountain Valley, CA 92708, USA; e Department of Psychiatry and Neuroscience, UC Riverside, Riverside, CA, USA; f Department of Neurology, Military Hospital 103, Vietnam Military Medical University, Ha Noi, Vietnam; g University Medical Center HCMC, University of Medicine and Pharmacy at HCMC, Ho Chi Minh City, Vietnam; h Department of General Internal Medicine, Hanoi Medical University Hospital, Ha Noi, Vietnam; i Department of Neurology and Alzheimer’s Disease, National Geriatric Hospital Vietnam, Ha Noi, Vietnam; j Stroke Center, Phu Tho General Hospital, Phu Tho Province, Vietnam

**Keywords:** Creutzfeldt-Jakob disease, sCJD, abnormal DWI or FLAIR, PSWCs

## Abstract

**Background:**

Accurate diagnosis of sCJD remains challenging in developing countries such as Vietnam, as clinical and research efforts focus on treatable diseases. Several cases of sCJD have been diagnosed in Vietnam but data from formal analyses are lacking. This is the first systematic analysis of patients with sCJD in Vietnam.

**Methods:**

This was a systematic retrospective review of medical records from patients with probable sCJD (N = 31) seen between April 2021 to April 2024 at four tertiary care centers. Clinical, laboratory, neuroimaging, and EEG findings were included in the analysis.

**Results:**

Data from 16 men and 15 women with sCJD were analyzed. The average age of onset was 63.4 years (range 50–83 years). Twenty-one of the patients had died after a mean survival of 7.6 months (range 3–20 months). All patients initially presented with rapidly progressive dementia. Other associated symptoms included myoclonus (77 %), extrapyramidal symptoms (80 %), pyramidal symptoms (58 %), akinetic mutism (55 %), visual disturbance (45 %), and cerebellar ataxia (32 %). Neuroimaging revealed abnormal fluidattenuated inversion recovery (FLAIR) and diffusion-weighted imaging (DWI) sequences in 28/31 patients. EEG revealed periodic sharp wave complexes (PSWCs) in 26/31 patients. Only 12 patients had been tested for 14–3-3 protein in CSF and all were positive.

**Conclusions:**

Clinical, neuroimaging, laboratory, and EEG features are consistent with global findings.

## 1 Introduction

Creutzfeldt-Jakob disease (CJD) is a fatal, neurodegenerative prion disease with an annual global incidence of approximately 1–2 million people [1,2]. CJD has a variable clinical presentation at onset and rapid progression, with a mean survival of 4 to 6 months [1,2]. Epidemiologic data suggest that the incidence of CJD may be on the rise, but it remains unknown whether this increase reflects a true rise in rates of disease or is related to factors such as aging of the population, better recognition, better diagnostic tools, or other factors [1,2]. Historically, diagnoses of CJD could only be made post-mortem, but more recent tools and methods have led to earlier diagnosis.

Complicating the early recognition and diagnosis of CJD is the fact that at least six distinct molecular subtypes have been identified, which vary in time of onset, survival, and neuropathological findings [1]. Sporadic CJD (sCJD) is the most common type of CJD, accounting for up to 85 % of cases [2]. Early diagnosis of sCJD can also be challenging due to its nonspecific clinical presentation and overlap with symptoms of other neurological and psychiatric conditions [3]. As a result, diagnosis often occurs when the disease is already advanced and the window for potential experimental therapies has closed [1].

Although sCJD has been extensively studied in many countries, in part with the assistance of surveillance infrastructure, its epidemiology is still poorly understood outside of North America, Europe, and some countries in Asia [4]. In Asia specifically, CJD is likely underreported in some areas due to regional disparities in surveillance and reporting [5]. Furthermore, data suggest that recognition and diagnosis are challenging and difficult to study in low- and lower-middle-income countries [4].

The goal of this retrospective descriptive analysis was to characterize patients with sCJD in Vietnam, which, to date, have largely been described in case studies. This research represents the first systematic report of the clinical, laboratory, neuroimaging, and electroencephalographic characteristics of patients with sCJD in Vietnam, utilizing data collected from four tertiary care hospitals. Better understanding of the initial and later presentation of these patients may help to improve diagnosis and recognition in this country.

## 2 Methods

### 2.1 Study design and patient population

This retrospective analysis was conducted on data collected from medical records of patients with sCJD treated at four tertiary care hospitals from April 2021 to April 2024: Bach Mai Hospital (Hanoi), Military Hospital 103 (Ho Chi Minh City), Hanoi Medical University Hospital, and University Medical Center Ho Chi Minh City. This study received ethical approval from the Biomedical Research Ethics Committee of Bach Mai Hospital.

Researchers at each medical center collected data from patients seen at their hospital using a customized research template. Electronic medical records were searched for diagnoses containing the key term “sporadic Creutzfeldt-Jakob Disease”. We found 36 patients with this diagnosis. However, only 31 patients met the United States Centers for Disease Control and Prevention 2010 diagnostic criteria for probable sCJD; a definitive diagnosis requires brain biopsy [6,7]. Criteria for probable sCJD include clinical manifestations of progressive dementia and at least two of the following four signs: myoclonus, visual or cerebellar disease, pyramidal or extrapyramidal symptoms, and akinetic mutism, as well as characteristic changes in electroencephalogram (EEG) and magnetic resonance imaging (MRI), or laboratory data, such as positive 14–3-3 protein in cerebrospinal fluid (CSF). Moreover, routine investigations must not suggest an alternative diagnosis. The remaining 5 cases identified by searching had missing information that precluded a diagnosis (MRI, EEG, lumbar puncture, or tests to rule out metabolic diseases). Of the final 31 cases, 18 were from Bach Mai Hospital, 8 were from the University of Medicine and Pharmacy Ho Chi Minh City, 2 were from 103 Hospital, and 3 were from Hanoi Medical University Hospital. A trained neurologist reviewed patient records and pertinent information extracted for the analysis.

### 2.2 Data collection and analysis

Baseline demographic, clinical, laboratory, MRI, and EEG data were abstracted from patient medical records and recorded on a custom research form. Specific clinical symptoms and signs associated with sCJD were collected from both subjective and objective portions of the medical record. Symptom onset was recorded as of the initial hospital visit. Symptoms after disease progression were recorded from the time of hospitalization to discharge. Results of CSF testing for protein 14–3-3 were available for 12 patients and were also recorded and included in the analysis. Survival time was defined as the time from disease onset until death. Follow-up information regarding deceased patients was obtained through phone calls with the patients' families.

Information was collected on cerebellar symptoms, defined as evidence of dysmetria on the finger-to-nose or heel-knee-shin test and nystagmus on eye exam; pyramidal signs, including hyperreflexia (positive Babinski sign or documented muscle spasticity); extrapyramidal signs including rigidity, tremor, bradykinesia, restless legs syndrome or dystonia; epilepsy based on clinical diagnosis with or without epileptiform spike-wave activity on EEG; neuropsychiatric symptoms of depression, fretfulness, anxiety, autism, apathy, insomnia, obsession, excitement, abnormal emotion, character change, abnormal behavior or memory disorder; and sleep disorders diagnosed clinically based on the presence of insomnia, lack of sleep, or hypersomnia. Polysomnography data was not available for any patients included in the analysis.

Data from two commonly used and well-validated assessments, the Mini Mental Status Exam (MMSE; [8]) and the Medical Research Council scale (MRC; [9]) were also collected and analyzed for this study. The MMSE is a 30-question clinician administered assessment of five areas of cognitive function: orientation, registration, attention, and calculation, recall, and language. Each question is rated as 0 or 1 for a maximum possible score of 30, with higher scores reflecting better cognitive function. A score of < 24 was considered indicative of dementia. Rapidly progressive dementia (RPD) was defined as onset of dementia within 1–=–2 years of disease onset [10]. The MRC is a milestone-based functional scale with both language and non-language components [9] used to evaluate dynamic aphasia characteristic of prion disease. The verbal component is a score of 0 (mute) to 4 (normal conversation), intended to identify gross onset and progression of language disturbance. An MRC score of < 4 was used to define the presence of a language disorder.

Objective findings included brain MRI, EEG, and lumbar puncture. All MRIs were performed using a 1.5-T superconducting magnet (GE Healthcare, Pittsburgh, PA, USA) and included T1, T2, fluid-attenuated inversion recovery (FLAIR), and diffusion-weighted imaging (DWI) sequences. All EEGs were performed using Nihon Kohden EEG-1200 K.

Descriptive data are reported. No statistical analysis was conducted.

## 3 Results

### 3.1 Demographic and clinical characteristics

Sixteen men and 15 women were included in this descriptive analysis. The mean age at disease onset was 63.4 years (range 50–83 years). Mean time to diagnosis of sCJD was 2.7 months (range 1–14 months) and mean time from diagnosis to death was 4.9 months (range 0.5–15.5 months). Twenty-one patients had been misdiagnosed at the primary hospital before transferring to the university hospital. Misdiagnoses included Parkinson's syndrome (n = 7), Alzheimer's disease (n = 6), psychosis (n = 4), encephalitis (n = 3), and epilepsy (n = 1).

Table 1 shows the most common symptoms at disease onset and following disease progression. The most common symptom at disease onset was RPD (84 %). Language disorders, extrapyramidal signs, visual disturbances, and neuropsychiatric disorders were reported in 29 % to 35 % of patients.

**Table 1:** Clinical symptoms at onset and after disease progression.

Characteristic	Number of patients (N = 31)
*Symptoms at onset*^a^	
Dementia (MMSE < 24)	26
Language disorders (MRC > 4)	26
Extrapyramidal symptoms	11
Neuropsychiatric symptoms	9
Visual disturbance	8
Vertigo, balance disorders	7
*Symptoms after disease progression*^b^	
Dementia (MMSE < 24)	31
Extrapyramidal symptoms	25
Myoclonus	24
Pyramidal symptoms	18
Akinetic mutism	17
Visual disturbance	14
Neuropsychiatric symptoms^c^	12
Cerebellar symptoms	10
Sleep disorders	9
Epilepsy^d^	8

MMSE, Mini Mental State Exam; MRC, Medical Research Council scale.

a *Onset symptoms are symptoms recorded as of the initial hospital day visit.*

b *Symptoms following disease progression were recorded from hospitalization until discharge.*

c *Neuropsychiatric disorders included hallucinations, delusions, and mood disorders.*

d *Two patients presented with both epilepsy and sleep disorders.*

Following disease progression, the most frequent clinical manifestations were continuing RPD (100 %), myoclonus (77 %), and extrapyramidal symptoms including parkinsonism and dystonia (81 %). Pyramidal symptoms, visual disturbance, and akinetic mutism were also common, as shown in Table 1. Other neurologic symptoms included cerebellar symptoms, epilepsy, neuropsychiatric symptoms, and sleep disorders were also noted (Table 1).

### 3.2 MRI and EEG findings

All patients included in this analysis had undergone MRI and EEG. Medical records review revealed that 28/31 patients (90 %) had hyperintensities on DWI and/or FLAIR sequences in the cortical and basal ganglia regions of the brain. In patients with hyperintensities, lesions were documented in the cortex in all 28 patients, the basal ganglia in 19 patients, or both (n = 19; Table 2).

**Table 2:** Key clinical, neuroimaging, and survival findings.

Findings	Patients
*Brain MRI*	*n = 31*
Abnormality (any), n	28
Cortical abnormality, n	28
Basal ganglia abnormality, n	19
DWI hyperintensity, n	26
FLAIR hyperintensity, n	28
*EEG*	*n = 31*
PSWC positive, n	26
Diffuse slow waves, n	16
Epileptiform discharges, n	14
*CSF*	*n = 31*
Elevated WBC count (>5 cells/µl), n	4
Elevated total protein (>0.5 g/l), n	5
*14*–*3-3 protein*	*n = 12*
Positive, n	12
Time of symptom onset in protein-positive patients, mean (range) weeks	10 (5–28)
*Survival*	*n = 21*
Survival time (time from onset to death), mean (range), months	7.6 (3–20)
Survival time ≤ 1 year, n	17

CSF, cerebrospinal fluid; DWI, diffusion-weighted imaging; EEG, electroencephalogram; FLAIR, fluid-attenuated inversion recovery; EEG, electroencephalogram; MRI, magnetic resonance imaging; PSWC, periodic sharp wave complexes; WBC, white blood cell.

Twenty-six patients (84 %) had evidence of periodic sharp wave complexes (PSWCs). Other abnormal EEG patterns included focal epileptiform discharges and diffuse slow waves as shown in Table 2.

In 3 patients with normal MRI findings, EEG showed PSWCs. In 5 patients with normal EEG, brain MRI revealed lesions.

**Fig. 1:**
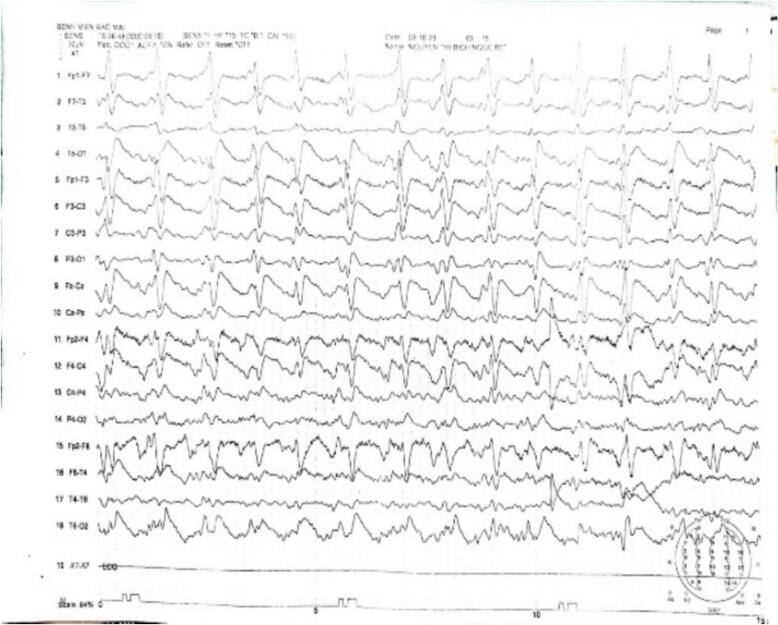
Electroencephalographic findings in a patient with probable sporadic Creutzfeldt-Jakob disease.

**Fig. 2:**
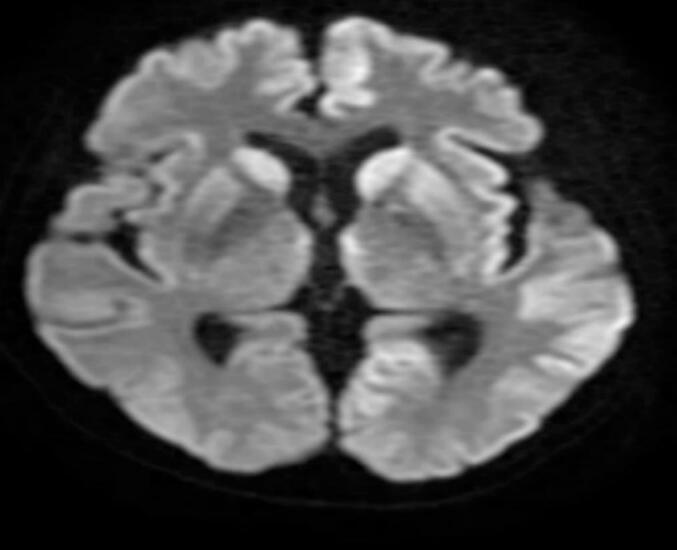
Diffusion-weighted MRI (DWI) shows hyperintensities in the bilateral caudate, putamen, and cortical regions in a Vietnamese patient with probably sporadic Cruetzfeld-Jakob disease.

Fig. 1, Fig. 2 show EEG and imaging findings, respectively, from a 61-year-old Vietnamese patient who presented with dementia, myoclonus, neuropsychiatric, pyramidal, and extrapyramidal symptoms. On hospital day 5, EEG revealed PSWCs on EEG and MRI DWI hyperintensities in the bilateral caudate, putamen and cortex.

### 3.3 CSF 14–3-3 protein

Twelve patients had data from CSF 14–3-3 protein testing, all demonstrating positive results. The mean time from symptoms onset to 14–3-3 protein positivity was 10 weeks (range 5–28 weeks; Table 2).

### 3.4 Survival

At the time we conducted this analysis, 10 patients were still alive. Twenty-one of the 31 patients in our analysis had died prior to our data extraction. Patients had a mean survival time of 7.6 months (range 3–20 months). Among these patients, 17 cases (55 %) survived < 1 year and 4 cases ≥ 1 year (Table 2).

## 4 Discussion

This retrospective, descriptive analysis of clinical symptoms, supportive diagnostic tests, and patient survival time provides the first comprehensive description of patients with sCJD in Vietnam. Results show that characteristics of patients with sCJD in Vietnam align with those expected based on diagnostic criteria and in most respects are similar to those reported for China [[11], [12]] and other parts of Asia [[13], [14], [15]], Europe [13], and the United States [14]. However, the relatively small number of patients identified in our analysis suggests that neither patients nor physicians are routinely able to identify early symptoms of disease. Further supporting that notion, 5 patients diagnosed with sCJD by their local neurologists did not, in fact meet CDC criteria for probable disease [6], and 21 of 31 patients were initially misdiagnosed.

There are number of possible reasons for delayed, incorrect, or missed diagnosis of sCJD in Vietnam. First, the number of neurologists practicing in Vietnam is approximately 0.48 per 100,000 people based on 2007 data [16]. Second, patients have difficulty accessing neurologists, who are often concentrated at the central hospital. Third, because of a cultural bias in Vietnam to focus limited medical resources on curable diseases, available neurologists are likely to be inadequately trained to identify sCJD or may not be looking for it. Finally, some primary care facilities have limited or no access to ancillary testing such as MRI, EEG, or CSF testing, which are required for probable diagnosis.

Definitive diagnosis of sCJD is even less likely in Vietnam, as it requires brain biopsy and neuropathological examination. Brain biopsy carries inherent risks and potential complications and are therefore generally unacceptable to Vietnamese patients given the fact that biopsy cannot inform or improve prognosis. Brain biopsies are commonly performed in Vietnam to help diagnose and treat brain tumors with the hope of positive outcome.

This study analyzing 31 patients with probable sCJD in Vietnam revealed the need for improved education and awareness about sCJD both so early symptoms can be monitored and to provide a more accurate estimate of the epidemiology of sCJD in the country. Our patients showed both similarities and differences compared to other countries. The average survival time for sCJD patients in Vietnam was shorter compared to those in China, the United States, and Japan (Table 3). This is likely due to patients being diagnosed later in the disease course when symptoms are more severe. Our study reported higher rates of PSWCs, MRI, and CSF 14–3-3 protein abnormalities compared to studies in China, Europe, and the United States (Table 3). The availability of advanced diagnostic methods, such as RT-QuIC and brain biopsy, was limited in this study. These tests should be more widely accessible and utilized to enhance diagnostic accuracy. In the interim, additional studies of patients in Vietnam should be conducted to continue to characterize patient characteristics and to determine whether surveillance data from other Asian countries may help to inform earlier and more accurate diagnosis.

**Table 3:** Regional comparison of epidemiology, patient demographics, diagnosis, and clinical presentation of sCJD

**Country**	**Our Study** **Vietnam (n = 31)**	**Eastern India** [15] **(n = 10)**	**Thailand** [17] **(n = 18)**	**Japan** [18] **(n = 674)**	**China**[11] **(n = 104)**	**China**[12] **(n = 67)**	**Europe** [13] **(n = 2451)**	**United States** [14] **(n = 116)**
***Demographics***								
Gender (% male)	52	40	44	43	54.8	71.6	45.7	67.2
Age of onset [mean (range) years]	64.4 (50–83)	56.1 (39–70)	66.7	66.7	60 (29–82)	64.42 (29–88)	67.2 (15.6–94.9)	65
Survival time [mean (range) months]	7.7 (3–20)	4.6	NR	15.7	9 (1–36)	9.39 (1–60)	5 (1–81)	10.6
Survival time ≤ 1 year (%)	80.9	NR	NR	NR	NR*	79.4	85.8	NR
Survival time > 1 year	19.1	NR	NR	NR	NR	20.6	14.2	NR
Initial misdiagnosis (%)	67.7	NR	NR	NR	56.7	NR	NR	NR
***Clinical symptoms (% of patients)***								
Rapid progressive dementia	100	100	100	NR	100	50.7	NR	95
Myoclonus	77.4	100	77.8	NR	NR	70.1	NR	63
Extrapyramidal	80.6	50	55.6	NR	37.2	NR	NR	65
Akinetic mutism	54.8	100	44.4	NR	45.2	37.3	NR	NR
Pyramidal	58.1	NR	55.6	NR	43.3	38.8	NR	NR
Visual disturbance	45.2	50	27.8	13.6	31.7	25.3	NR	64
Cerebellar ataxia	32.2	40	50	NR	51.9	70.1	NR	81
Epilepsy	25.8	NR	NR	NR	NR	NR	NR	NR
Neuropsychiatric	38.7	60	33.3	NR	7.7	58.2	NR	41
Sleep disorders	29	NR	44.4	NR	4.8	NR	NR	NR
***Key testing features***								
Periodic sharp wave complexes (%)	83.8	60	66.7	91.9	67	46	58.4	35
Abnormal MRI (%)	90.3	100	94	NR	88.2	84.8	39.1	64
Cortex (%)	90.3	NR	38.9	NR	82.4	75.7	NR	31
Basal ganglia (%)	61.3	NR	66.7	NR	38.2	75.7	NR	51
CSF 14–3-3 protein positive (%)	100	NR	NR	NR	34.1	68	88.1	71
Time of symptom onset to 14–3-3 protein positive (mean, weeks)	10 (5–28)	NR	NR	NR	NR	NR	NR	21.4
Bisophy (%)	0	NR	NR	NR	9.6	NR	NR	16
Autophy (%)	0	NR	NR	NR	1	NR	NR	NR

NR=not reported

## CRediT authorship contribution statement

**Khoi Hong Vo:** Writing – original draft, Supervision, Resources, Methodology, Investigation, Formal analysis, Data curation, Conceptualization. **Nga Thi Bui:** Writing – original draft, Methodology, Investigation, Formal analysis, Data curation. **Dung Thi Hoang:** Writing – review & editing, Writing – original draft, Resources, Methodology, Investigation, Formal analysis, Data curation, Conceptualization. **Thanh Vinh Nguyen:** Writing – original draft, Resources, Investigation, Data curation. **Binh Thanh Nguyen:** Writing – original draft, Supervision, Resources, Methodology, Investigation, Formal analysis, Data curation, Conceptualization. **Tai Ngọc Tran:** Writing – original draft, Resources, Investigation, Data curation. **Anh Hai Nguyen:** Writing – original draft, Resources, Investigation, Data curation. **My Thi Le:** Writing – original draft, Resources, Investigation, Data curation. **Ha Thi Do:** Writing – original draft. **Thuan Duc Nguyen:** Writing – original draft, Resources, Investigation, Data curation. **Hai Van Ta:** Writing – original draft, Resources, Investigation. **Toan Van Phan:** Writing – original draft, Resources, Investigation, Data curation. **Phuc Duy Phan:** Writing – original draft, Resources, Investigation, Data curation. **Thang Xuan Pham:** Writing – original draft, Resources, Investigation, Data curation. **Frank Xing:** Writing – review & editing, Writing – original draft, Resources, Investigation, Data curation. **Daniel Truong:** Writing – review & editing, Writing – original draft, Supervision, Resources, Methodology, Investigation, Formal analysis, Data curation, Conceptualization.

## Declaration of competing interest

The authors declare that they have no known competing financial interests or personal relationships that could have appeared to influence the work reported in this paper.
